# Exploring the Novel Input Attributes Affecting eWOM

**DOI:** 10.3389/fpsyg.2020.02017

**Published:** 2020-08-21

**Authors:** Safdar Hussain, Kaishan Huang, Zahida Ilyas, Ben Niu

**Affiliations:** ^1^College of Management, Shenzhen University, Shenzhen, China; ^2^Pir Mehr Ali Shah, Arid Agriculture University Rawalpindi, Rawalpindi, Pakistan; ^3^Great Bay Area International Institute for Innovation, Shenzhen, China; ^4^Department of Applied Psychology, Lahore College for Women University, Lahore, Pakistan

**Keywords:** online opinions, information-related interaction, information usefulness, purchasing behavior, WOM and eWOM

## Abstract

Electronic word of mouth (eWOM) has become significantly important in online communities, which are influential sources of instant information on the internet. This study examines the eWOM input attributes linked to consumers’ information adoption behavior in the context of information-related interaction. This study uses a structural equation modeling approach by choosing participants from Fujian and Guangdong provinces of China. The results reveal that eWOM input attributes studied positively influence information-related interactions. An individual’s perception of value enhances the performance of products or services, which is an essential predictor of information adoption. Furthermore, information usefulness and related interactions are key eWOM message characteristics affecting information adoption on the internet. This study contributes to the eWOM input attributes and message characteristics literature by exploring information-related interaction as a new mediator to consumers’ online information adoption. The authors provide suggestions for marketers and firms to dynamically develop strategies in response to consumers’ concerns while making a purchase online.

## Introduction

Traditional retailers are developing e-commerce platforms to pay attention to market growth and attracting purchasers. The importance of the internet cannot be undermined because internet users participate in online communities directly or indirectly, which is a vital form of electronic word of mouth (eWOM) (Gvili and Levy, 2016, p. 1030–1051; Mohammadi et al., 2019, p. 21). Word of mouth (WOM) has expanded into eWOM, such as electronic bulletins, newsgroups, blogs, online discussion forums, reviews, and networking sites. On these forums, everyone can share their experiences and knowledge about products and services with others, which provide credible information to the consumers (Kim and Lee, 2019, p. 163–176). Traditional conversation about products and services with colleagues, friends, and family members has transformed into eWOM, influencing consumer’s purchasing behavior greatly (Moalosi et al., 2006, p. 1–6). Now, customers consult online discussion forums before making a decision about a product or service (Hennig-Thurau et al., 2004, p. 51–74; Weinberg and Davis, 2005, p. 1609–1621). Since 2004, word of mouth (WOM) has expanded development in marketing, while eWOM became attractive for practitioners and researchers because of the advancement and significant growth in e-commerce (Hussain et al., 2020, p. 1–13).

People’s behavior during online decision-making processes has changed due to the increased use of the internet to seek information and opinions from social networking platforms to make the right decision about purchasing a product or service (Bickart and Schindler, 2001, p. 31–40; Hennig-Thurau et al., 2004, p. 38–52; Ho and Wang, 2015, p. 18). Researchers have observed that there is limited literature available on consumers’ eWOM intention, and there is a need to know whether the spread of positive eWOM via online platforms has some influence on traditional conversations (Ho and Wang, 2015, p. 18). This study tries to address the gap of whether customers depend on the exchange of information on the internet positively or not and to evaluate motivational involvement in eWOM that can influence a consumer’s information adoption behavior. Further, this study investigates the social phenomenon of eWOM behavior, adopted from the study of Sussman and Siegal (2003). Previous studies have focused on specific industries such as automobile, hospitality, tourism, DVDs, movie, computer software, books, and digital cameras. However, very few studies have examined information adoption behavior through eWOM communication (Hussain et al., 2018, p. 22–32, 2020, p. 1–13).

This study represents the first attempt to link the gap to find the indirect eWOM effects through the mediation role of information-related interaction associated with information usefulness that may influence consumers’ information adoption behavior. We aim to explore the objectives; this construct will enhance the values of performance of an individual’s perception and could help consumers to make better decisions. To build a helpful marketing strategy, marketers need to understand the eWOM information adoption effect, to determine not only direct eWOM input attributes but also indirect mediated relationship.

**FIGURE 1:**
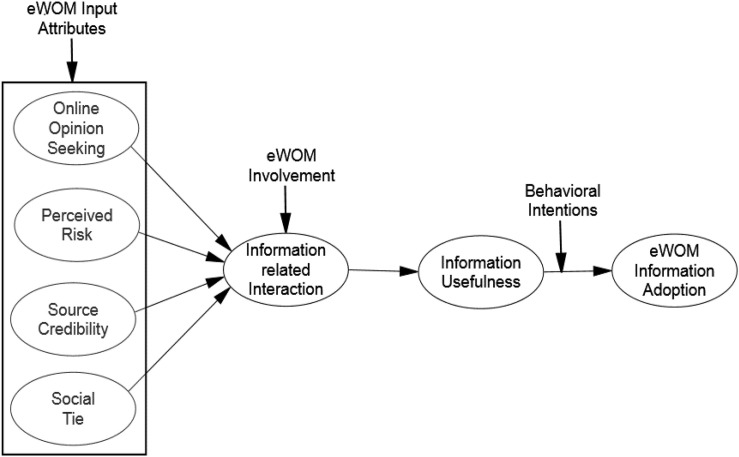
Proposed research model.

Our survey found that constructive input attributes play a decisive role in information perception and consumers’ behavioral intentions. Our results contribute to the existing body of consumers’ behavior literature by providing numerous managerial insights into the efficaciousness of products and services’ motives on consumers’ intentions. This specific research aims to contribute to the literature by filling a gap in the literature to elaborate the study’s generalizability of the association between purchase motives on the behavior of consumers through the mediating influence of cross-industry panel data sets. This model is the first to investigate the relationship between eWOM input attributes and consumers’ behavior through a mediating role of information-related interaction (Li et al., 2019, p. 3579). Figure 1 shows the conceptual framework; there are seven variables formulated by the above literature. Based on the literature, this study proposed a model with the following objectives:

• To analyze critical elements of customers’ motives linked to perception development.
• To examine the eWOM input attributes affecting information-related interaction for information adoption.
• To address the online customers’ information adoption behavior for products and services on online platform.

## Literature Review for Hypothesis Development

China has emerged as a significant global consumer market because of economic restructuring, raised living standards for individuals, enhanced prosperity, and innovative practices in retailing initiated by economic reforms by the state in 1978 (Cui and Liu, 2001, p. 84–1061; Arnett, 2002, p. 774–783; Xiao and Kim, 2009, p. 610–624; Hussain et al., 2019, p. 1144). In the first half of 2008, consumer spending in China reached over 5,104 billion RMB, which equaled 730 billion US dollars, according to the report by China’s National Statistics Bureau in 2008. As a result, numerous foreign firms initiated entry into Chinese consumer markets offering an explosion of product categories (Zhou and Hui, 2003, p. 36–58; Zhan et al., 2017, p. S2919–S2934). Triandis (2000) described that eWOM plays a significant role in consumers’ decision making, as this process of information gathering is an antecedent to individuals’ psychological processes (Triandis, 2000, p. 145–152). There likely are literature gaps and a lack of understanding of Chinese consumer markets (Marsh and Li, 2018, p. 124–137; Leal Filho and Rayman-Bacchus, 2019, p. 277–287), which is essential for understanding product- and service-related factors that drive demand.

Consumers are increasingly turning to online shopping sites, and eWOM has been found as an influential source for consumers’ purchase intentions. It is interesting to know whether anonymous people have more influence on consumers’ purchase intentions than those who are already known by the consumer. Evidence suggests that the ideas exchange from customer to customer (C2C) has a direct relationship with consumers’ behavioral intentions and an indirect mediated relationship through firms’ value, which is a form of eWOM (Tien et al., 2019, p. 238–249). eWOM consists of one of the anonymously useful information sources that facilitate the consumers-to-peer experiences, knowledge acquisition, and opinions through a variety of online platforms, discussion forums, blogs, and review websites (Daugherty and Hoffman, 2014, p. 82–102; Saura et al., 2020, p. 1–14). Likewise, eWOM attributes such as online opinion seeking, eWOM directions, perceived risk, source credibility, and social ties are the major antecedents that affect information adoption directly or indirectly (Park and Lee, 2009, p. 61–67; Erkan and Evans, 2018, p. 617–632; Reyes-Menendez et al., 2019, p. 68868–68877). Reyes-Menendez et al. (2020), p. (1–11) identified the influence of new technologies on behavioral issues of users in terms of the “#MeToo” movement, a social identity movement closely linked to the influence of individuals who reveal their thoughts in society for a better understanding, with positive and negative connotations supported by the communication adoption (Fujita et al., 2018, p. 55–71). Luo et al. (2014, p. 446–456) and Gvili and Levy (2019, p. 1–16) also argued that individual orientation tends to be more self-reliant and independent to make judgments supported by the perceived ease of use and usefulness to predict individual attitudes and intentions that explain information adoption.

### eWOM Input Attributes

eWOM input attributes include the reader’s motivations such as online opinion seeking, perceived risk, source credibility, and social ties (Hussain et al., 2020, p. 1–13). On online forums, opinion leaders provide consumption-related advice to receivers. Opinion leaders are considered more influential and credible than a commercial source, that is why marketers are interested to know how traditional WOM may differ from eWOM communication. Marketers want to know how to develop new strategies that can influence opinion seeking and how to hire influential opinion leaders who can be a positive spokesperson for them (Hennig-Thurau et al., 2004, p. 38–52; Tsang and Zhou, 2005, p. 1186–1193; Huete-Alcocer, 2017, p. 1–4). In a virtual community, opinion leaders are like public bulletin boards and may influence consumer behavior (Kotler, 2000, p. 188–193). Information need and advice seeking are motivators for eWOM engagement that affects consumers’ buying behavior.

Opinion seekers tend to seek a piece of advice or information from others to make a decision. Perceiving the risk in a situation because of no prior experience or familiarity with the product, they prefer to seek advice or information from others for better decisions (Hennig-Thurau et al., 2003, p. 51–74; Khammash and Griffiths, 2011, p. 82–87).

Perceived risk is a consumer’s feelings about unpleasant consequences and uncertainty because of several alternatives available toward making a purchase (Kim et al., 2005, p. 188–193). Hussain et al. (2017, p. 96–102) considered risk as a subjective judgment of results with the possibility to influence consumer behavior. Source credibility is the perceived trust in the information source by the receiver. Expertise and trustworthiness are two significant factors to evaluate source credibility. However, objectivity and homophily are also dimensions that can influence consumers’ purchasing behavior (Wiener and Mowen, 1986, p. 1–8). Ohanian (1990, p. 39–52) validated that information given by experts with authority, specific knowledge, competence, website reputation, skillfulness, and qualification has more influence and positively affects the receivers’ attitude to reduce the perceived risk of a purchase. Information receivers believe that popular online forums or review sites, number of reviews, registered reviewers, etc. provide them with helpful information. Another critical determinant of eWOM source credibility is trustworthiness, which may positively affect a receivers’ trust. Being active participants who share knowledge quickly and frequently on online forums resulted in an increase in trust. Consumers believe that the actual information, reviewer’s experience, reviewer’s effort, honest reviews, and length of contents validate trust (Xie et al., 2011, p. 178–183).

Social tie refers to a receiver’s perception about the quality of a product based on the viewers’ emotions and biases. Reviews written by the same gender, age, social class, interests, and similar buying patterns lead to homophily, which reduces the uncertainty of risk during the decision-making process (Hussain et al., 2017, p. 96–10).

***H1*:** eWOM input attributes, such as online opinion seeking, source credibility, and social ties have a positive influence on information-related interaction, whereas perceived risk has a negative impact on information-related interaction.

### Information-Related Interaction

eWOM information adoption behavior is a vital element of behavioral intention development, and researchers have recently discussed how resources may sustain the identity and diversity of eWOM information adoption in online communities (Chang et al., 2013; Chai et al., 2015; Horlings, 2015). However, the problem is how eWOM information adoption has influenced consumers’ perceptions shaped by society. Information-related interaction can lead to a positive adaptation activated by traditions that occur in threatening circumstances and challenges (Daskon and Binns, 2009, p. 494–517). Many studies, such as those by Holling (1973, p. 1–23); Barthel-Bouchier (2016, p. 53–78); Cerquetti and Ferrara (2018, p. 774), argued that the external physical applications of policymaking maintain capital constancy, while the resilient relationship as a construct of internal perspective represents an information system that addresses the concerns of behavioral intention vitality.

***H2*:** Information-related interaction has a positive influence on information usefulness.

### Information Usefulness and eWOM Adoption

An individual’s perception of value enhances the performance of a product or service, which is an essential predictor of adoption (Cheung et al., 2008, p. 229–247; Chuang et al., 2016, p. 6). Online opinions and ideas expressed on computer-generated platforms could help consumers make better decisions. Sussman and Siegal (2003, p. 47–65) argued that information usefulness is an effective predictor of intention or adoption because consumers take part in online communities to receive helpful information. Consumer confidence has a significant and considerable influence on adopting comments found to be useful. Information adoption is knowledge transfer that can influence people’s behavior to consider the nature of argument quality and source credibility centrally or peripherally driven from the information adoption model (IAM) (Sussman and Siegal, 2003, p. 47–65). IAM typically consists of four components of information transfer: argument quality (central route), source credibility (peripheral way), information usefulness, and information adoption. Sussman and Siegal (2003, p. 47–65) extended the knowledge of IAM by integrating the information adoption process with the dual process of the elaboration likelihood model (ELM) (Ajzen, 1991, p. 179–211).

***H3*:** Information usefulness has a significant impact on information adoption.

## Materials and Methods

### The Data Collection Procedure

The model of this study has introduced three factors in its proposed framework. In this specific model, the independent variables are the eWOM input attributes (online opinion seeking, perceived risk, source credibility, and social ties), which leads information-related interaction (a mediating variable) toward eWOM consumers’ behavioral intention (a dependent variable). This research study used a five-point Likert scale to assess the performance of the selected factors for this study, ranging from 1 for strongly disagree to 5 for strongly agree (Van Laerhoven et al., 2004, p. 830–835; Croasmun and Ostrom, 2011, p. 19–22). In the first step, the authors reviewed this study in depth. In stage 2, we designed and tested a pilot study for obtaining a clear and sound understanding of the selected items’ suitability for the questionnaire and adjusted the scale accordingly (Christianson et al., 2019, p. 659–669). In the final phase that is stage 3, the authors conducted the survey and recorded feedback from the recruited respondents (Phillips and Stawarski, 2016, p. 96–109).

### Questionnaire Designing

In designing a questionnaire, the authors developed the scale according to the study’s framework and distributed the adjusted version of the questionnaire among recruited respondents for collecting desired data. The authors educated and trained respondents about the study’s purpose and assured them that all the received data would be strictly confidential. The questionnaire contained two sections on the respondents’ perception of traditional eWOM input attributes (online opinion seeking, perceived risk, source credibility, and social ties), with consumers’ perception (information-related interaction) as the mediating variable toward eWOM consumers’ behavioral intention, which is a dependent variable. This research study also received general information on the participants such as age, gender, education, designation, and residential areas in the first section. In the second section of the questionnaire, the study addressed influencing and critical factors. This prospective study invited recruited participants from the selected population to fill the questionnaire forms (Krosnick and Fabrigar, 1997, p. 141–164).

This designed scale used a five-point Likert scale expecting respondents to rate the agreement level, by representing “strongly disagree” as 1 and “strongly agree” as 5 for measuring the performance of the selected elements of the study (Joshi et al., 2015, p. 396–403; Krosnick, 2018, p. 439–455). The nature of this study examination is causal research, in a non-contrived setting to minimize the extent of interference. The study invited 466 willing respondents for their inputs to examine the stated hypotheses while using a non-probability sampling technique. To analyze the results, the study selected individual participants and collected cross-sectional data from internet users who used online platforms to adopt information (Looi, 2005, p. 3). The study examined data reliability and validity through SPSS Cronbach’s alpha and confirmatory factor analysis for the appropriation of multivariate data, as proposed by Thompson (2004). In this study, structural equation modeling (SEM) was used for model fitness because SEM is being used as a technique to combine and measure complex path models in social work research (Ullman and Bentler, 2003, p. 830–835; Majeed et al., 2020, p. 599).

### Sample Size, Data Processing, and Feedback

The sample size of this specific population is 651, and the authors distributed the designed questionnaire to the selected respondents through a designed survey in the Fujian and Guangdong provinces of China. The authors received 466 complete/accurate responses from the selected participants. After receiving the filled forms, the authors examined and screened all the responses of the survey. Based on accurate and duly answered responses, they scrutinized these forms to confirm the accuracy of the data collected. This proposed model of the study processed all the received feedback and analyzed the data by applying SPSS and structural equation modeling (SEM) tools. In the final stage of data processing, the statistical analysis showed the interpreted findings as practical insights and valuable results.

### Research Instruments

The eWOM input attributes, such as online opinion seeking, perceived risk, source credibility, and social ties were measured using the paradigms of Bettman (1973, p. 184–190); Hennig-Thurau et al. (2004, p. 38–52); Kim et al. (2005, p. 188–193); Sun et al. (2006, p. 1104–1127); Park and Lee (2008, p. 386–398); Chu and Kim (2011, p. 47–75); Khammash and Griffiths (2011, p. 82–87); Toder-Alon et al. (2014, p. 42–64); Wang et al. (2018, p. 54–62). The characteristics of human-centered development perception, such as information-related interaction, were measured from authors self-developed research items using paradigms of Erkan and Evans (2016, p. 47–55); Hussain et al. (2018, p. 22–32, 2020, p. 1–13). This study adopted information usefulness and information adoption from the earlier studies of Wu and Shaffer (1987, p. 677) and Citrin (2002, p. 1–143). This research also used the works of Sussman and Siegal (2003, p. 47–65); Erkan and Evans (2016, p. 47–55); Hussain et al. (2018, p. 22–32) by applying the elaboration likelihood model (ELM), as presented by Sussman and Siegal (2003, p. 47–65) to analyze the consumers’ engagement in online communities.

## Results

**FIGURE 2:**
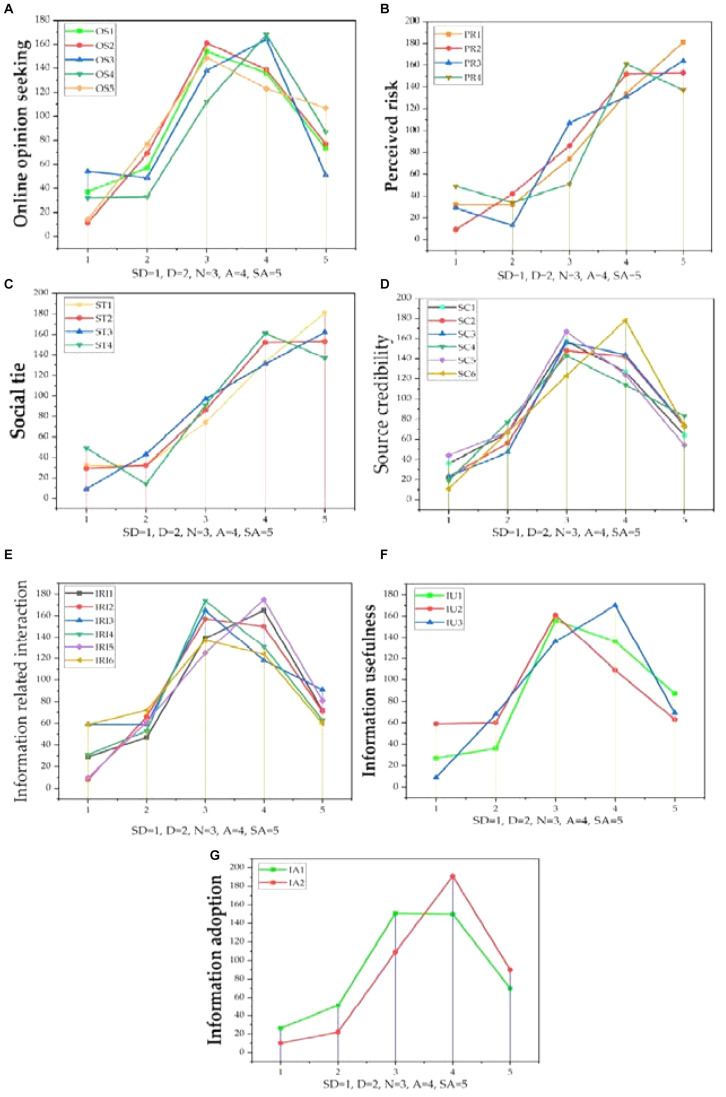
Graphical presentation of each variable **(A–G)** indicating the rate of respondents’ willingness (strongly disagree to agree strongly) (*n* = 466).

The results of this research provided support for the arguments on the intended behavior of Chinese consumers to adopt online information. Table 1 presents that out of 466 respondents, 47% (219) were male, while 53% (247) were female. Sixty-eight percent (317) of the participants were between the ages of 18 and 40 years. Seventy-two percent (335) either had a diploma/certificate or held a bachelor’s degree. Forty seven percent of the participants were between the monthly income brackets of 5001 and 10,000 RMB. Ninety-one percent of the participants had used the internet before, and 9% respondents had not. Eighty-nine percent of the respondents had shopped online previously. The average frequency of online shopping of the respondents was one to five times in a month, while 67% of the participants bought online before purchasing decisions. The previous literature witnessed that most of Chinese consumers prefer to read online comments or reviews before purchase. Eighty-seven percent (405) read reviews or comments on online forums before making a decision. Online consumers were asked to rate their willingness to adopt online information; for this, the Likert scale was used to measure from strongly disagree to strongly agree for the said purpose. Figure 2 shows a graphical presentation of each variable indicating the respondents’ willingness (strongly disagree to strongly agree).

**TABLE 1:** Respondents’ demographic information (*n* = 466).

Categories		Frequency	Percentage
Gender	Male	219	47.0
	Female	247	53.0
Age	18–30	162	34.8
	31–40	155	33.2
	41–50	107	23.0
	51 and above	42	09.0
Education level	Higher secondary school or lower	49	10.5
	Diploma/certification	187	40.1
	Bachelor degree holder	148	31.8
	Postgraduate degree holder	83	17.6
Monthly income	3,000 RMB or lower	66	14.2
	3,001–5,000 RMB	108	23.2
	5,001–10,000 RMB	219	46.9
	10,001 RMB or above	73	15.7
Have you	Yes	314	67.3
shopped online before?	No	81	20.6
Read reviews	Yes	347	87.8
or comments	No	48	12.2

The psychometric properties of constructs explained the validity and reliability of the instrument. The authors characterized and examined reliability of the data received by using SPSS Cronbach’s alpha and conformity factor analysis (CFA). Table 2 shows the values of Cronbach’s alpha (α), average variance extracted (AVE), and discriminant validity (DV), which present acceptable values with the ranges of each hypothesis. The outcome of each factor presented a decent value such as online opinion seeking (α = 0.824, *AVE* = 0.80, *DV* = 0.912), perceived risk (α = 0.919, *AVE* = 0.69, *DV* = 0.906), social ties (α = 0.921, *AVE* = 0.64, *DV* = 0.887), eWOM source credibility (α = 0.881, *AVE* = 0.65, *DV* = 0.910), information-related interaction (α = 0.939, *AVE* = 0.63, *DV* = 0.916), information usefulness (α = 0.845, *AVE* = 0.81, *DV* = 0.936), and information adoption (α = 0.792, *AVE* = 0.66, *DV* = 0.765). Table 2 presents the factor loading values of 31 research items indicating the instrument validity of each construct.

**TABLE 2:** Psychometric properties of constructs (*n* = 466).

Construct	Items	Factor loadings	Construct	Items	Factor loadings
Online opinion seeking (α = 0.824, *AVE* = 0.80, *DV* = 0.912)	OS1	0.744	Source credibility (α = 0.881, *AVE* = 0.65, *DV* = 0.910)	SC1	0.830
	OS2	0.712		SC2	0.818
	OS3	0.893		SC3	0.813
	OS4	0.883		SC4	0.770
	OS5	0.854		SC5	0.824
Perceived risk (α = 0.919, *AVE* = 0.69, *DV* = 0.906)	PR1	0.892		SC6	0.767
	PR2	0.824	Information related interaction (α = 0.939, *AVE* = 0.63, *DV* = 0.916)	IRI1	0.828
	PR3	0.898		IRI2	0.771
	PR4	0.745		IRI3	0.828
Social tie (α = 0.921, *AVE* = 0.64, *DV* = 0.877)	ST1	0.800		IRI4	0.733
	ST2	0.864		IRI5	0.814
	ST3	0.830		IRI6	0.821
	ST4	0.710	Information usefulness (α = 0.845, *AVE* = 0.81, *DV* = 0.936)	IU1	0.892
Information adoption (α = 0.792, *AVE* = 0.66, *DV* = 0.765)	IA1	0.867		IU2	0.910
	IA2	0.754		IU3	0.893

**α, Cronbach’s alpha; DV, discriminant validity; AVE, average variance extracted.**

**FIGURE 3:**
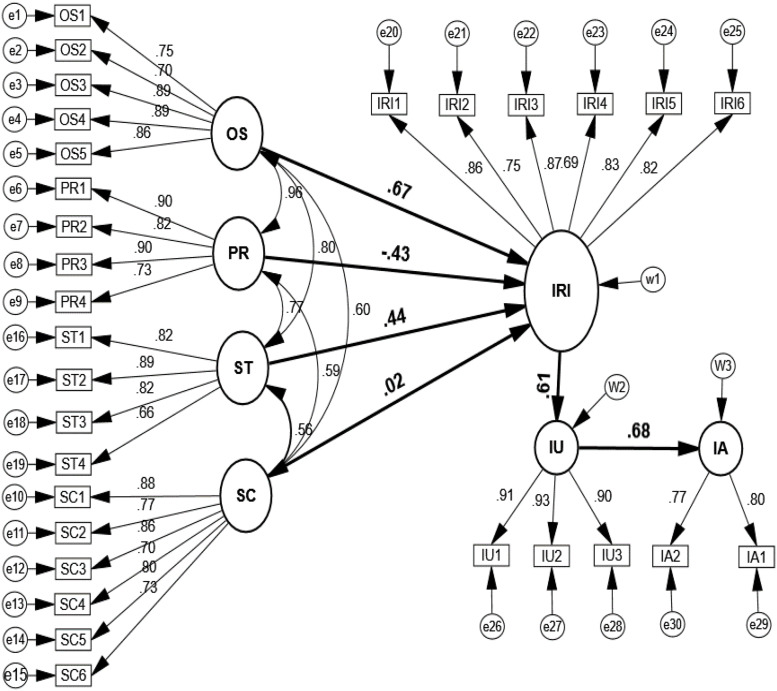
Structural equation model. OS, online opinion seeking; PR, perceived risk; SC, source credibility; ST, social tie; IRI, information-related interaction; IU, information usefulness; IA, information adoption.

The structural model shows the relationship between the variables. The *p*-value is indicated at.000 because the *p*-value presents the appropriate assumptions for model fitness in the population entirely (Beauducel and Herzberg, 2006, p. 186–203). The acceptable values of chi-square/df range from 1 to 3, as recommended by Bryant and Satorra (2012, p. 372–398). The chi-square/df for this model was 2.387, which indicated a better fit for the conceptual model. The benchmark values of the comparative fit index (CFI) and Tucker–Lewis index (TLI) were.951 and.964, respectively, and are accepted, whereas the goodness of fit values index (GFI) and goodness-of-fit index (AGFI) are.918 and.927, respectively, which are slightly acceptable. Besides, the value of the root mean square of approximation (RMSEA) is.059, which presented a fair value (Hair et al., 1998, p. 627–677) (see Figure 3).

Table 3 shows that the estimated weight of regression coefficient for different causal paths indicated that online opinion seeking, source credibility, and social ties supported information-related interaction with estimated values of 0.675, 0.017, and 0.438, whereas the perceived risk did not support information-related interaction with an estimated value of −0.433. Information usefulness was supported by information-related interaction. The estimated value of the causal paths was 0.605, and information usefulness with an estimated value of 0.676 had supported information adoption accordingly (see Table 3).

**TABLE 3:** The standardized regression weights of causal paths (*n* = 466).

Casual paths			Estimates	S. Est.	SE	CR	Results
IRI	< —	OS	0.571	0.675^∗∗∗^	0.259	2.205	Supported
IRI	< —	PR	−0.349	−0.433	0.229	−1.524	Not Supported
IRI	< —	SC	0.014	0.017	0.05	0.286	Supported
IRI	< —	ST	0.426	0.438^∗∗∗^	0.094	4.554	Supported
IU	< —	IRI	0.785	0.605^∗∗∗^	0.074	10.666	Supported
IA	< —	IU	0.508	0.676^∗∗∗^	0.048	10.606	Supported

**S. Est, standard estimates; SE, standard errors; CR, critical ratio; ^∗∗∗^p < 0.05.**

## Discussion

This study attempted to develop the anticipated research area and designed a proposed model to bridge the likely gap on eWOM input attributes by conducting an in-depth review of the previous research literature. This study proved our hypotheses on online opinion seeking, social ties, and source credibility by showing that these factors have a positive impact on information-related interaction, whereas perceived risk has a negative influence on information-related communication. Online opinion leadership is an essential variable in an online community, and consumers consider them as the first person to try the products and regard them as leaders. Opinion leaders believe that their community thinks about them as a source of information (Kim et al., 2015; Zhou et al., 2019, p. 36–58). They consider themselves an excellent advice-giving source of information online for others. In this study, online idea seeking refers to searching for others’ comments, ideas, and latest online information and seeking advice from friends through chat rooms, web reviews, and emails. Online opinion seekers consult other people’s opinions to feel more comfortable with their buying decision and seek positive or negative reviews on websites before making the purchase decision. Social ties developed a strong/weak mechanism that influences information-related interaction, which affects consumer purchase intentions. Consumers feel that reading reviews helps to reduce ambiguity, to decrease the unpleasant experiences, and to increase their confidence during decision making (Zhang and Khare, 2009, p. 524–537; Erkan and Evans, 2016, p. 47–55; Hussain et al., 2017, p. 96–102).

Source credibility is an observation of a receiver who believes that several reviews provided by popular forums and review sites help deliver information and knowledge and predict experience and usefulness. Receivers believe that trustworthiness reflects the actual level of studies, reviewer’s expertise, the honesty of the reviews, and reviewer’s effort. Source credibility also relies on reviews written by those people who are of the same gender and age and have similar interests and purchasing patterns (Bickart and Schindler, 2001, p. 31–40; Xie et al., 2011, p. 178–183; Hussain et al., 2017, p. 96–102).

The hypotheses on information-related interaction having a positive impact on information usefulness is supported by the results. The degree of reviews has a weak relationship with information usefulness because people consider that the information provided by others is not informative, valuable, and helpful for decision making. Lastly, the variable information adoption positively affects information usefulness because consumers follow opinions as well as agree with suggestions given in the comments about a product or service on the internet.

### Theoretical and Practical Implications

This study contributes to the eWOM input attributes literature through the proposed relationships between mediated predictions such as information-related interaction, and information usefulness was used to analyze the acceptance of consumers’ eWOM information adoption within online network communities. Our expectations that information-related interaction can predict the results also confirmed eWOM information adoption. It has been observed that consumers follow opinions and agree with suggestions given in the comments about a product or service on the internet. From this, it can be concluded that information-related interaction may influence consumers’ behavior because consumers feel that reading reviews helps to reduce ambiguity, decrease the unpleasant experiences, and increase their confidence during decision making. It is very much unlikely for all of the consumers to be satisfied, and the reason is a weak relationship with information usefulness and degree of reviews because people consider that the information provided by others is not informative, valuable, and helpful for decision making. Notable among many of the issues faced by online marketers is the need to focus explicitly on developing possible consequences associated with eWOM input attributes and information-related interactions’ characteristics to attain business growth and capture consumer attention in a saturated and dynamic environment. Marketers and online companies must understand all of the consequences and antecedents of an individual’s perception to engage online consumers effectively and build confidence during the decision making that may have a significant and considerable influence on adopting comments.

## Conclusion

eWOM plays an essential role in online communities and influences consumer’s information adoption behavior. The notion of this study was to develop a theoretical framework and provide new insights into the current literature on online communication. Previous research studies documented that eWOM input attributes influence purchasing decisions. Thus, eWOM message characteristics contribute within the context of comments and reviews that lead to adopting online information provided by online communities because of information-related interaction. Based on the results, this research determined that online opinion seeking and source credibility are critical elements of consumers’ intention associated with eWOM input attributes. By addressing the gap, this study found that eWOM source credibility is an essential characteristic of the eWOM message for online information adoption.

There is an absolute demand for future studies to examine social dimensions of eWOM input attributes primarily to highlight the results in response to social interaction and communication, which has a positive impact on purchasing decisions. The findings provide useful information for consumers of product or services, firm owners, and brand managers for addressing issues such as improving services which could enhance a firm’s competitiveness to attain business growth and profitability. To increase the perception of eWOM input attributes, marketers need to focus explicitly on developing appropriate website features and functions to attract customers. Relevant information can increase the consumers’ interest in using the reviews effectively. The limitations we faced are associated with the homogeneity of the population used for this study since the group consisted of respondents from Fujian and Guangdong provinces of the Republic of China. We also did not investigate online perception that can be influenced by other products and countries as well. This study regards overall consumers’ predictions about online shopping websites and product or services rather than focusing on one website or a specific product or service type. Future research could focus on one specific online shopping website and product or service type as well through the current findings by adding this model. The current study investigated some eWOM input attributes because of limited time and resources. We consider other eWOM input attributes such as uncertainty, experience, prior knowledge, information giving, and information seeking as useful for future business of consumer products. This research focused on eWOM message characteristics, however, eWOM information processing, eWOM system, and eWOM platforms need to be compared as eWOM process attributes in the future.

## Data Availability Statement

The raw data supporting the conclusions of this article will be made available by the authors, without undue reservation, to any qualified researcher.

## Ethics Statement

The studies involving human participants were reviewed and approved by the Board of Studies and Research of College of Management, Shenzhen University, Shenzhen, China. The patients/participants provided their written informed consent to participate in this study.

## Author Contributions

SH developed the conceptual notions and drafted and revised the manuscript. KH and ZI contributed to the literature, methods, and conclusion. BN reviewed the revised the manuscript critically, provided substantial contributions, and approved the final version to be submitted. All authors contributed to the article and approved the submitted version.

## Conflict of Interest

The authors declare that the research was conducted in the absence of any commercial or financial relationships that could be construed as a potential conflict of interest.
